# Book Notices

**Published:** 1899-04

**Authors:** 


### BOOK NOTICES.

Ovariotomy Averted is the title of a pamphlet of ten pages. By Dr. Mary J. Hall-Williams, of "The Nook," Penzance, Cornwall, England. Price, 2 pence.

In this essay the author denounces the operation of excising the ovaries, and argues for a more humane and rational treatment of ovarian troubles with medicines and an ingenious uterine support invented by herself.

There is no doubt of the truth of the statements made, and the pamphlet will convince any one who may read it.

The Rational Treatment of Acute and Chronic Diseases of the Genito-urinary Tract. By Charles Marchand, Chemist and Graduate of the "Ecole Centrale, des Arts and Manufactures de Paris."

This is a pamphlet of forty pages, detailing the result of treating certain cases with hydrozone and peroxide of hydrogen. These are gonorrhœa, gleet, urethritis, cancer and gangrene of uterus, pelvic abscess, recto-vaginal fistula, endometritis, protracted labor, chancres, and chancroids. All these conditions are reports by prominent physicians detailing their methods of using hydrozone and their experience in treating cases with it. To this end clinical cases are given and the whole book is a clear exposition of the essential nature of hydrozone, peroxide of hydrogen, and glycozone and methods of application. Copies may be had free of charge by addressing a note to Mr. Charles Marchand, at his *new* address, 57 and 59 Prince Street, New York.

